# Interleukin-37 reduces lipopolysaccharide induced matrix metalloproteinase-9 in gingival epithelial cells

**DOI:** 10.1186/s12903-025-06016-z

**Published:** 2025-04-25

**Authors:** Arzu Beklen, Muhammet Burak Yavuz, Deniz Uckan

**Affiliations:** 1 Translational Immunology Research Program (TRIMM), Research Program Unit (RPU), University of Helsinki, Helsinki, Finland; 2 Department of Periodontology, Faculty of Dentistry, Eskisehir Osmangazi University, Eskisehir, Turkey; 3 Bogazici University, Medico-Social Dental Clinic, Istanbul, Turkey

**Keywords:** Epithelial cells, IL-37, LPS, MMP-9, Periodontitis

## Abstract

**Background:**

In periodontal diseases, the recognition of pathogen-associated molecular patterns (PAMPs) triggers signaling cascades that lead to the release of matrix metalloproteinases (MMPs). Interleukin-37 (IL-37) is recognized as a key suppressor of the immune response. This study aimed to detect the expression and distribution of IL-37 in gingival tissues and analyze its suppressor role in MMP-9 in response to lipopolysaccharide (LPS)-stimulated gingival epithelial cells.

**Methods:**

Immunohistochemistry localized IL-37 in gingival tissues from periodontitis patients and healthy controls (*N* = 10). The induction of IL-37 expression by LPS was analyzed using the conditioned medium of gingival epithelial cells through enzyme-linked immunosorbent assay (ELISA). To determine the relevant MMP-9 levels in epithelial cells following exposure to LPS alone or in combination with IL-37, both quantitative PCR (qPCR) and enzyme-linked immunosorbent assay (ELISA) were performed.

**Results:**

Cultured epithelial cells secreted significantly higher levels of IL-37 when stimulated with LPS compared to unstimulated controls. Both ELISA and qPCR showed that LPS stimulation significantly increased MMP-9 levels. However, co-culture with IL-37 markedly reduced LPS-induced MMP-9 expression at both the protein and mRNA levels. Furthermore, immunohistochemistry revealed increased IL-37 expression in periodontitis tissues, both in epithelial cells and connective tissue.

**Conclusions:**

Gingival epithelial cells may contribute to tissue responses in periodontitis through the secretion of MMP-9 in response to PAMPs. Furthermore, IL-37 appears to have a potential role in modulating and reducing this response, as observed in the decreased MMP-9 expression following IL-37 co-stimulation.

## Background

Periodontitis is a prevalent chronic inflammatory disease affecting the supporting structures of the teeth and is primarily driven by a dysbiotic microbial community within the subgingival biofilm [1]. The transition from a symbiotic to a dysbiotic state is influenced by microbial shifts, environmental factors, and host immune responses, leading to sustained inflammation and tissue destruction [2, 3]. Dysbiosis is characterized by an increase in keystone periopathogens, which modulate the host immune response by impairing antimicrobial clearance while promoting chronic inflammation [4]. The activation of pattern recognition receptors (PRRs), including Toll-like receptors (TLRs) and Nod-like receptors (NLRs), by pathogen-associated molecular patterns (PAMPs) triggers downstream signaling cascades, leading to the production of pro-inflammatory cytokines and matrix metalloproteinases (MMPs) that contribute to extracellular matrix degradation and alveolar bone resorption [5, 6]. This persistent immune dysregulation ultimately disrupts periodontal homeostasis, promoting disease progression and tissue destruction.

MMPs are calcium dependent, zinc containing endopeptidases capable to degrade components of the extracellular matrix [7]. Gelatinase B, MMP-9, is a member of a subfamily of MMPs that degrades collagen and is one of the most abundant MMPs in periodontal tissues reflecting periodontal disease severity [8]. MMP-9 is produced by many cell types, including gingival epithelial cells. Furthermore, MMP-9 is an important component in many biological and pathological processes because of its ability to directly degrade extracellular matrix proteins and to activate cytokines and chemokines to regulate tissue remodeling in periodontal tissues [9]. We have previously demonstrated a significant association between the pro- and active forms of MMP-9, suggesting a critical role for MMP-9 in the degradation and turnover of periodontal tissue components during disease progression [10].

Stimulation of gingival epithelial cells with bacterial products initiates a cascade of proteases and the degradation of the extracellular matrix in the tissue. Gingival epithelial cells not only play an important role as a mechanical barrier against bacterial invasion but also act as the primary secretory cellular lineage of the innate immune response to infectious inflammation in periodontal tissue [11]. Toll-like receptor (TLR) 4 was the first toll-like receptor in humans to be characterized, and it is located on the cell surface of epithelial cells [12, 13]. TLR-4 recognizes various PAMPs, like bacterial lipopolysaccharide, along with several other components of pathogens, leading to chronic inflammation in periodontal tissue [14].

Compared to other cytokine families, the interleukin (IL)–1 family has a central role in triggering key signaling molecules that contribute to the pathogenesis of periodontitis [15]. IL-1 exerts different effects on different cells [16], primarily influencing innate immunity but also playing a role in adaptive immunity [17]. As a recently identified member of the IL-1 family, IL-37 suppresses innate inflammation and inflammatory responses in different diseases, such as Graves’ disease [18] and cardiac dysfunction [19]. Although IL-37 expression has been detected in different human tissues, such as the liver, lung, thymus, bone marrow, lymph nodes, and placenta [20], only a quite low level of expression was found in healthy skin tissues [21]. Furthermore, low levels of IL-37 in the peripheral blood mononuclear cells of healthy individuals were significantly upregulated after PAMP engagement [22].

Limited studies have focused on the role of IL-37 in periodontitis, yielding diverse conclusions. In this regard, higher IL-37 expression was found in both the epithelial layer and connective tissue layer of gingival tissue samples from periodontitis patients [23]. In another study, clinical data demonstrated a decrease in IL-37 levels in the gingival crevicular fluid samples of periodontitis patients but failed to differentiate healthy patients from those with periodontal disease [24].

Therefore, understanding the impact of in vitro exposure to PAMPs on gingival epithelial cells will allow us to analyze the functional aspects of IL-37 in periodontal tissues. Due to the complexity of the periodontal microbiota and the diversity of the host response, it remains unknown whether IL-37 displays an immunoregulatory role in periodontal disease and how it reacts to infiltrating microorganisms. In this study, we aimed to characterize MMP-9 release triggered by LPS under the influence of IL-37 in gingival epithelial cells. The null hypotheses tested were as follows: There is no difference in IL-37 expression between healthy and periodontitis tissues, and IL-37 has no effect on immunological changes.

## Methods

### Patients and samples

This study complied with the Declaration of Helsinki (2002) and was approved by the Bogazici University Ethical Committee (2019/015). Gingival tissue samples were obtained with informed consent from periodontitis patients (*n* = 10, age 38–49) during routine flap surgery and from healthy controls (*n* = 10, age 23–38) during wisdom tooth extraction. All participants were systemically healthy, non-smokers, and had not received periodontal therapy or antibiotics/anti-inflammatory drugs in the previous six months. Pregnant/lactating women and individuals with habits affecting periodontal health were excluded. The age difference between groups may influence immune responses; however, all participants were systemically healthy, non-smokers, and free from confounding factors. Periodontitis is primarily driven by microbial dysbiosis and immune dysregulation rather than age alone [1, 2]. The study design minimized potential biases, ensuring that observed differences were disease-related [25, 26].

Full-mouth periodontal examinations assessed gingival index (GI), pocket depth (PD), clinical attachment level (CAL), bleeding on probing (BOP), and plaque index (PI). Periodontitis patients had ≥ 20 teeth, ≥ 4 teeth per jaw, PD ≥ 5 mm, GI ≥ 2, CAL ≥ 4 mm, ≥ 50% bone loss in at least two quadrants, and BOP > 80%. Healthy controls had ≥ 20 teeth, PD ≤ 3 mm at ≥ 90% of sites, GI ≤ 1 at ≤ 15% of sites, and no radiographic bone loss.

Tissue samples were collected, washed with PBS, fixed in 10% formalin, dehydrated, cleared in xylene, and embedded in paraffin. The slides were dewaxed, rehydrated, and stained with haematoxylin and eosin [27] prior to incubation with specific IgG.

### Immunohistochemical staining

Slides (6 μm) were deparaffinized, rehydrated, and subjected to antigen retrieval using citrate buffer in a microwave. Endogenous peroxidase activity was blocked with 0.3% H_2_O_2_ in methanol, followed by PBS washes. Non-specific binding was blocked with horse serum (1:50 in PBS with 5% BSA). Samples were then incubated overnight with rabbit anti-human IL-37 IgG (1:1000, Thermo Fisher). After PBS washes, biotinylated anti-rabbit IgG and an avidin-biotin-peroxidase complex (Vectastain ABC Kit) were applied. Color development was carried out using H_2_O_2_ and diaminobenzidine, after which slides were counterstained, dehydrated, and mounted. The immunohistochemical staining was conducted under standardized conditions to ensure reproducibility and reliability of the findings. Non-immune rabbit IgG was used as a control.

### Cell culture and cytokine stimulation on gingival epithelial cells

Human gingival epithelial cells were isolated via explant culture and maintained in keratinocyte growth medium 2 (Dulbecco’s modified Eagle’s medium, Gibco, Life Technologies, Paisley, UK). Cells were incubated at 37 °C with 5% CO2, with medium changes every 5 days. At ∼ 80% confluence, cells were subcultured (1:3), and passages 2–4 were used. For experiments, 2 × 10⁶ cells/well were seeded in six-well plates and incubated in serum-free medium for 2 days before stimulation with LPS (10 ng/mL) and/or IL-37 (50 ng/mL, R&D Systems Inc., Minneapolis, MN, USA). After 24 h, the culture medium was collected and stored at -80 °C. We confirmed their epithelial identity through both morphological and molecular approaches. The cells exhibited a characteristic cobblestone-like morphology under phase-contrast microscopy. Additionally, qPCR analysis demonstrated strong expression of epithelial markers, including KRT19 (Cytokeratin 19), a well-established epithelial marker; ZO-1 (Zonula Occludens-1), a tight junction protein indicating epithelial barrier function; and E-Cadherin (CDH1), an adhesion molecule specific to epithelial cells, further validating their epithelial nature. Experiments were performed in triplicate with three biological replicates, and IL-37 and MMP-9 levels were analyzed via ELISA and/or qPCR.

### Enzyme-linked immunosorbent assay of interleukin-37 and matrix metalloproteinases-9

IL-37 and MMP-9 production from gingival epithelial cell cultures were measured using Quantikine ELISA kits (R&D Systems Inc., Minneapolis, MN, USA) according to the manufacturer’s instructions. The detection limits were 31 pg/mL for IL-37 and 0.2 ng/mL for MMP-9. Standards and samples were added to antibody-coated wells, followed by incubation with biotinylated antibodies. After washing, a substrate solution containing hydrogen peroxide and tetramethylbenzidine was added, and the reaction was stopped with a stop solution. Absorbance was measured at 450 nm using a spectrophotometer.

### Quantitative Real-Time PCR

Total RNA was extracted using the RNeasy Plus Mini Kit (Qiagen) and subsequently converted to cDNA using the iScript cDNA Synthesis Kit (Bio-Rad). Quantitative real-time PCR (qPCR) was performed with 10 ng of cDNA per reaction using the LightCycler 480 SYBR Green I Master Mix (Roche) on the LightCycler 96 system (Roche). The primers used for MMP-9 were: forward 5’-CGCAGACATCGTCATCCAGT-3’ and reverse 5’-GGATTGGCCTTGGAAGATGA-3’. Relative gene expression was determined using the 2^(-ΔΔCt) method, with RPLP0 (housekeeping gene) forward 5’-GAAATCCTGAGTGATGTGCAGC-3’ and reverse 5’-TCGAACACCTGCTGGATGAC-3’.

### Evaluation of immunostaining

Randomly selected images were captured at 20× magnification. The number of stained cells was counted in three randomly selected fields per section and expressed as the mean ± SEM per mm². The intensity of staining was categorized as weak (+), moderate (++), or strong (+++). To interpret the results, moderate and strong cytoplasmic diffuse staining with distinct nuclear staining were considered.

### Statistical analysis

Statistical calculations were performed using GraphPad Prism version 5.00 data analysis program (GraphPad Software, Inc., La Jolla, California, USA). Data were expressed as the mean ± SEM. Before applying statistical tests, the normality of the data was assessed using the Shapiro-Wilk test. For normally distributed data, one-way analysis of variance (ANOVA) followed by Dunn’s multiple comparison test was used to compare the differences among the mean values of different groups. *P*-values < 0.05 were considered statistically significant. For comparisons between two groups, Student’s t-test was used. The number of biological replicates was 3, with 3 technical replicates for each in vitro experiment.

## Results

Table 1 shows the clinical parameters of the sites from which gingival tissue samples were collected. Between the healthy and periodontitis affected group, there were not any significant differences in gender or age (*p* > 0.05). The clinical parameters were significantly higher in the periodontitis affected group than in the healthy group (*p* < 0.01), reflecting the severity of periodontal tissue destruction.

**Table 1:** Clinical peiodontal parameters for healthy and periodontitis patients

Clinical Parameter	Healthy Patients	Periodontitis Patients
PI*	10.75 ± 1.13	83.13 ± 2.69
GI*	0.15 ± 0.07	2.68 ± 0.19
PD*	2.38 ± 0.18	6.20 ± 0.81
CAL*	0 ± 0	3.92 ± 0.30
BOP*	12.4 ± 0.98	84.20 ± 2.28

Data are means ± SEM. *Significantly different from periodontitis group. PI, plaque index; GI, gingival index PD, probing depth (mm); CAL, clinical attachment level (mm); BOP, bleeding on probing

### Immunohistochemical staining of the gingival tissues with interleukin-37

Most of the IL-37 immunoreactive cells were found in the epithelium in the healthy tissue samples. In contrast, IL-37 was expressed in epithelial cells, endothelial cells, and in fibroblast- and macrophage-like cells in the lamina propria. The apparent number of IL-37 positive cells and their staining intensity were high in the diseased tissue samples compared with the healthy tissue samples.

The number of IL-37 expressing cells was similar in patients with periodontitis and healthy controls in the superficial epithelial layer. In contrast, cells expressing IL-37 showed higher frequency in the basal epithelial cell layer in periodontitis samples compared with healthy samples. The common finding in all periodontitis samples was that IL-37 expressing cells showed increasing staining intensity in periodontitis samples compared to the healthy group.

**Fig. 1:**
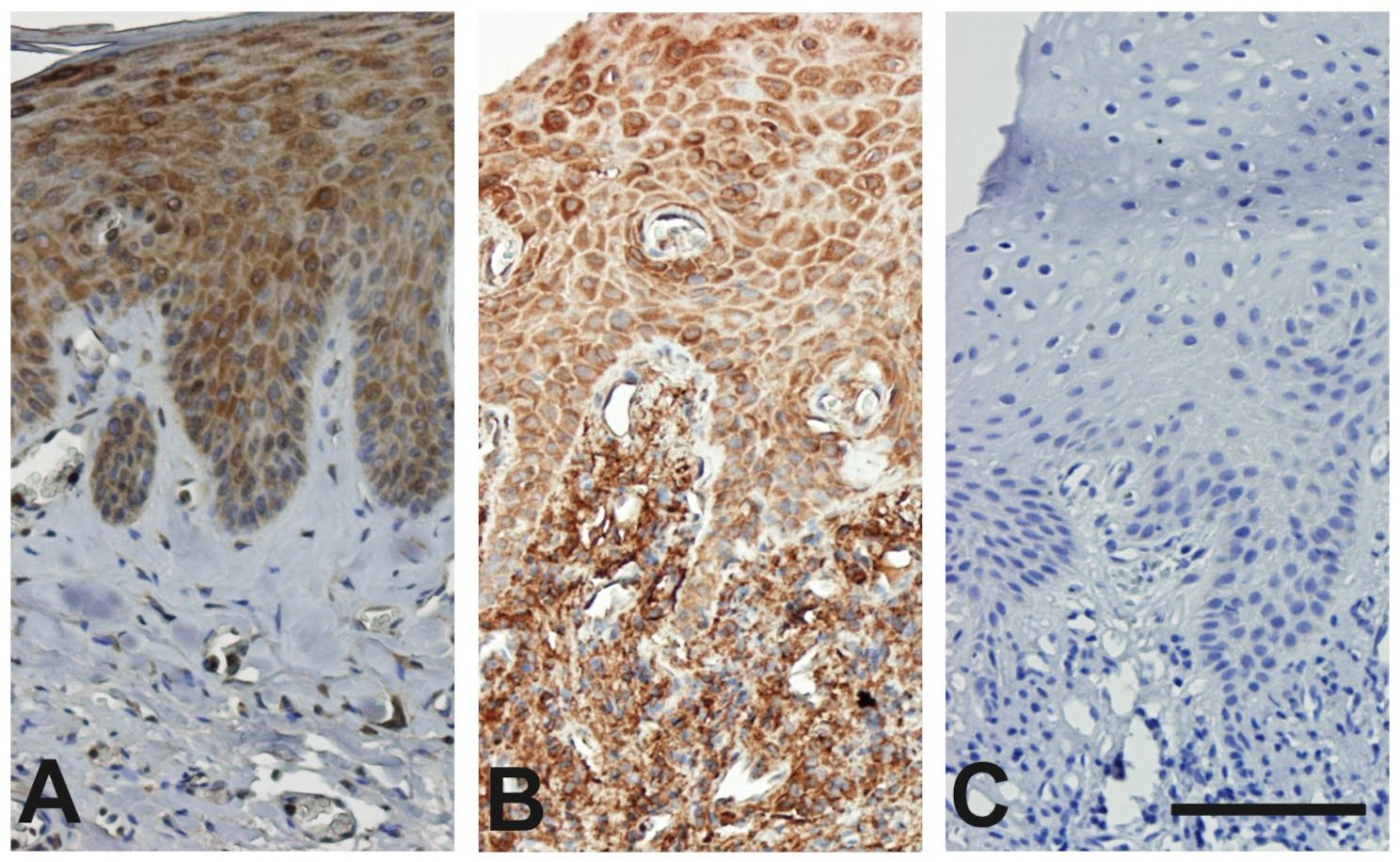
Representative pictures of interleukin 37 (IL-37) staining in the gingival tissues were (**A**) in healthy gingiva with IL-37 was less frequently expressed, whereas (**B**) intense and more frequent IL-37 immunoreactivity was seen in periodontitis effected tissues and (**C**) no staining is detected in the negative staining control. IL-37 is localized in epithelial cells, monocyte/macrophage-, fibroblast-like, and vascular endothelial cells. Scale bar = 100 μm

The semiquantitative analysis revealed increased immunoreactivity in the epithelial cells and lamina propria of the periodontitis-affected samples compared with the healthy tissue samples (417.1 ± 26.36 vs. 315.6 ± 17.31, *p* < 0.01), and (931.10 ± 79.66 vs. 144.40 ± 9.38, *p* < 0.001), consequently (Fig. 1).

### Induction of IL-37 in human gingival epithelial cells upon stimulation with lipopolysaccharides

**Fig. 2:**
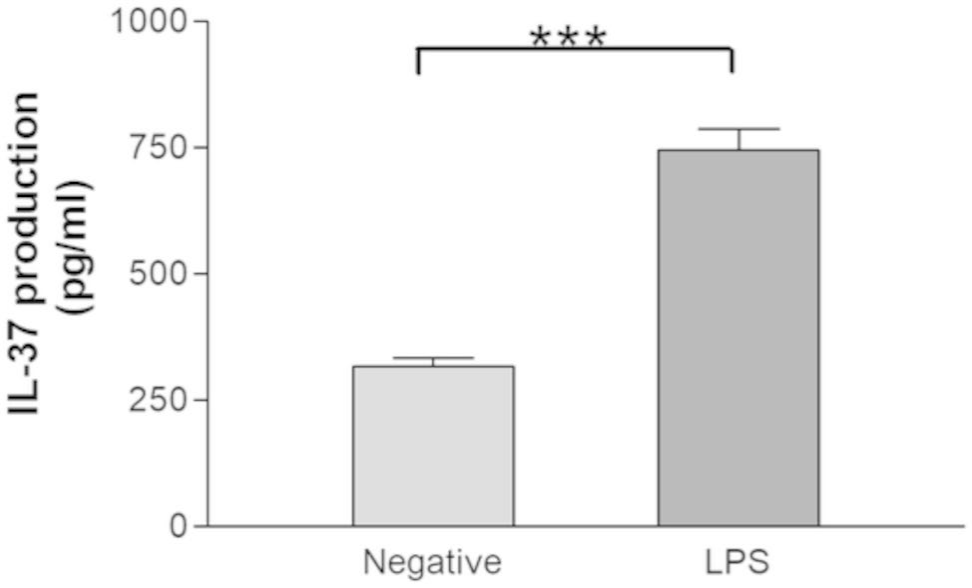
Effect of LPS induction on IL-37 concentration in gingival epithelial cell cultures in vitro by ELISA. Mean ± standard error of mean is shown. Lipopolysaccharides (LPS) and interleukin (IL). The results are from triplicate measurements, for both non-stimulated/negative and stimulated cultures. ***p < 0.001, analyses by t-test

The effect of LPS stimulation on gingival epithelial cells showed that such stimulation significantly increased IL-37 production compared with non-treated cells (746.30 ± 41.38 vs. 317.30 ± 16.32, *p* < 0.001) (Fig. 2).

### Matrix Metalloproteinase-9 gene expression in gingival epithelial cells, by qPCR

**Fig. 3:**
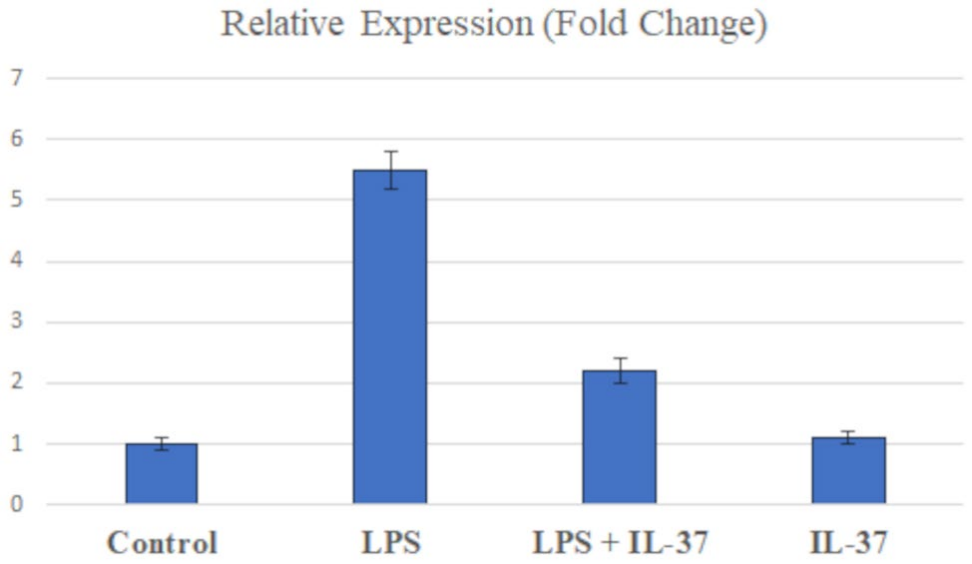
Relative MMP-9 gene expression in gingival epithelial cells. qPCR analysis showed LPS significantly increased MMP-9 expression, while IL-37 co-stimulation reduced this effect. IL-37 alone resulted in expression levels similar to the control. Data are mean ± SE from three independent experiments

The impact of LPS and/or IL-37 co-stimulation on *MMP-9* gene expression is presented in Fig. 3. qPCR analysis revealed that stimulation with LPS markedly upregulated *MMP-9* gene expression with a fold change of 5.5 ± 0.3 compared to the untreated control group (1.0 ± 0.1). However, co-stimulation with IL-37 significantly attenuated the LPS-induced increase in *MMP-9* transcript levels, with a fold change of 2.2 ± 0.2 compared to the LPS-only treated group (5.5 ± 0.3). Notably, cells treated with IL-37 alone exhibited *MMP-9* expression levels comparable to those observed in the non-stimulated control group, with a fold change of 1.1 ± 0.1 (Fig. 3).

**Fig. 4:**
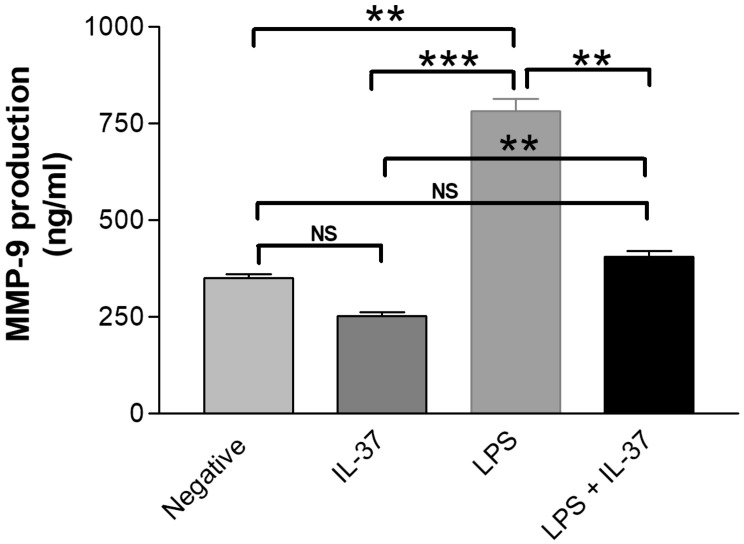
Effect of LPS and/or plus IL-37 on induction of MMP-9 concentration in gingival epithelial cell cultures in vitro by ELISA. Mean ± standard error of mean is shown. Lipopolysaccharide (LPS), interleukin (IL) and matrix metalloproteinase-9 (MMP-9). The results are from triplicate measurements, for non-stimulated/negative and stimulated cultures. Negative (NEG). NS: not significant, **p < 0.01, ***p < 0.001, analyses by ANOVA

### MMP-9 expression in response to LPS with and without IL-37, by Enzyme-linked immunosorbent assay

Compared with non-challenged cells, LPS upregulated the expression of MMP-9 in epithelial cells (349.90 ± 9.93 vs. 782.40 ± 31.13, *p* < 0.01). The co-stimulation of IL-37 under the effect of LPS decreased MMP-9 production by two-fold compared with single LPS treated cells (782.40 ± 31.13 vs. 405.4 ± 14.58, *p* < 0.01). However, the observed downregulation remained higher compared to single IL-37 stimulated cells (251.7 ± 9.62) (*p* < 0.01) (Fig. 4).

## Discussion

The study aimed to analyze how IL-37 responds in periodontal disease and its biological significance in gingival epithelial cells during inflammation. We demonstrated that IL-37 was expressed with higher immunoreactivity intensity and a greater frequency of positive cells in diseased patients. Furthermore, we showed that epithelial cells, by reducing the tissue-degrading enzyme MMP-9, mitigating inflammation severity, and maintaining homeostasis, recruit IL-37 in response to LPS stimulation. The null hypothesis was rejected.

In periodontitis-affected tissues, IL-37 expression extends beyond the epithelial layer into the underlying connective tissue, suggesting an altered regulatory role in response to inflammation. While immunohistochemistry may not reveal extreme differences within the epithelial layer, IL-37’s broader distribution highlights its potential role in the inflammatory microenvironment. Given the active involvement of gingival epithelial cells in immune modulation and MMP-9 production, investigating IL-37’s effects in these cells remains a scientifically valid approach.

The initiation of periodontal disease depends on a complex interaction between the microbial challenge of dental biofilm and the host response [28]. The pocket around a tooth forms a suitable ecological niche for dental biofilm with more than 700 bacterial presenting different bacterial cell constituents [29]. Among these cell constituents, the LPS has been proposed in various ways to interact with the inflammatory response. In periodontal diseases, the most attention for immune modulation has been directed toward LPS, because LPS stimulates cells to secrete certain proinflammatory cytokines, which can induce other cytokine cascades leading to severe forms of periodontal disease. Since, LPS is a very important immunoreactive molecule and seen as a molecular pattern by gingival epithelial cells, in this current study LPS is used as a stimulant [28, 30].

Recent studies highlight the critical role of antimicrobial peptides (AMPs) in oral innate immunity, where they regulate microbial communities and inflammation alongside cytokines like IL-37. For instance, AMPs such as cathelicidin LL-37 and defensins exhibit broad-spectrum activity against oral pathogens while modulating immune responses [31]. Similarly, AMP-coated dental implants demonstrate reduced bacterial adhesion and biofilm formation, underscoring their therapeutic potential in periodontal disease [32].

This study demonstrated that IL-37 is expressed in both the epithelial cell layer and the connective tissue of healthy and diseased gingival tissues. In periodontal disease, IL-37 expression was significantly higher in connective tissue compared to epithelial cells. The relatively smaller increase in epithelial expression appears to help maintain host homeostasis against biofilm organisms under healthy conditions. The functional basis for this pattern is the presence of pattern-recognizing Toll-like receptors (TLRs) on the surface of epithelial cells [33]. A straightforward explanation for this is that in healthy niches, adjacent to the epithelial surface, the continuous exposure to bacterial constituents activates specific TLRs, thereby triggering innate immune responses aimed at maintaining homeostasis [34]. In this context, our findings align with those of Jing et al., who assessed IL-37 expression in both healthy and diseased periodontal tissues using immunohistochemistry [23].

In clinically inflamed periodontal tissues, a higher bacterial burden creates an anaerobic niche (i.e., LPS in periodontal pockets) activates the lining epithelial cells and mononuclear inflammatory cells, such as macrophages and lymphocytes, start to migrate to underlying connective tissue associated with the increased disease severity [35]. Our findings confirmed and extended those reports that resident and migrating cells participate actively in inflammatory processes by expressing IL-37 [23]. IL-37 upregulation is likely because new cells recruited into connective tissue to respond via local cellular proliferation and recruitment from the circulation [36]. Our conclusion is based on the high number and intense staining of the IL-37-positive producer cells in periodontitis tissue samples and LPS treated epithelial cells. Once the toll-like receptors are stimulated by LPS, not only the epithelial cells but also the cells of connective tissue elicited signals resulting in the production of IL-37 cytokine microenvironment [37]. Unlike most members of the IL-1 family, IL-37 is an anti-inflammatory cytokine and we showed that IL-37 limits downstream inflammation, namely by suppressing MMP-9, in gingival epithelial cells [38]. Because IL-37 is a marker of cellular activation, we revealed that both epithelial layer and connective tissue participate actively in the local inflammatory processes.

Apart from disease typical LPS-induced MMP-9 production, the co-stimulation of IL-37 markedly decreased MMP-9 production in epithelial cells. MMPs are considered to be associated with tissue turnover or periodontal tissue degradation dependent on increased levels [9]. In parallel to our study, an excessive amount of MMP-9 production and activation were analyzed and linked to severe types of periodontal disease [39]. The findings of these previous studies were in accordance with the current finding that MMP-9 was found both in healthy and diseased samples and the expression was higher in disease-typical PAMP (LPS) stimulated samples compared to controls [40]. LPS regulator-profile was exhaustively studied in many studies. Toraya et al. have speculated that bacterial LPS stimulated synthesis and release of MMP-9 via NF-κB activation [41], and interestingly NF-κB activation was found to be significantly increased in the situation, in which IL-37 was silent in human coronary artery endothelial cells [42]. This corresponds well with our observations revealing gingival epithelial cells presenting evidence of a significant decrease of LPS induced MMP-9 under the effect of IL-37.

The increase of IL-37 mRNA and protein amount in the inflammation process has a capacity to stabilize the transcription of IL-37 [43]. In our diseased samples the observed higher IL-37 expression level might indicate that, the cells overexpressed the anti-inflammatory cytokine to protect the tissue from the metabolic consequences of chronic inflammation, which in turn contribute to even more severe clinical parameters. A general consensus on the effect of IL-37 is that an increased amount of IL-37 level is critical to block other central pro-inflammatory cytokines [38, 44] and apparent levels of IL-37 lead to pathological changes in tissues [45]. The more extensive recruitment of IL-37 in periodontitis might indicate the extra burden posed by periodontal inflammation. Correspondingly, the study of Nold et al. reported that elevated IL-37 amount suppressed the LPS stimulation to block pro-inflammatory microenvironment as we observed in the current study [22]. Worth mentioning, in periodontal diseases, dynamic changes in the level of LPS and consequently cytokines such as IL-1 beta and TNF-alpha results an increased level of the collagen degrading enzymes MMP-9 [46]. Notably, the present work clearly demonstrated that IL-37 acted as a regulator cytokine to maintain an anti-inflammatory stage and exhibited a certain protective effect through the regulation of MMP-9 in gingival epithelial cells.

The detected certain amount of MMP-9 activity was critical for normal epithelial turnover in our unstimulated cells similar to a study of Franco and coworkers [47]. However, in LPS triggered cells, the excessive active MMP-9 was an indicator of ongoing inflammation and sign of extracellular matrix breakdown leading to the pathology of periodontal tissue destruction [9]. In fact, the suppression of excessive MMP-9 via elevated IL-37 levels might be an interesting new therapeutic approach in periodontal diseases. Although LPS-induced cytokines or signaling kinases are reduced in the human cell lines following the transfection with IL-37 [48], the inhibition of MMP-9 can not be simplified only to the direct an autocrine mechanism such as downregulating via IL-37 secretion without considering the effect of tissue inhibitors of metalloproteinase (TIMP) expression [49]. As PAMP treatment increases the balanced ratio level of MMP-9 to its inhibitors, the proteolytic activity of MMP rapidly regulates the inflammatory cell recruitment into the vessel wall. Furthermore, due to the highly complex nature of cytokine regulation in extracellular matrix degradation, the expression of MMPs and TIMPs presents various disparate processes in each cell type. Such as, in the presence of IL-10, which is another anti-inflammatuar IL-1 family member, it acts to downregulate MMP-9 expression but stimulates TIMP-1 expression in monocyte/macrophage [50]. In light of this, the biological capacity of IL-37 on MMP-inhibitors would be interesting to study in gingival epithelial cells under pathological conditions.

## Conclusion

We conclude that epithelial cells and connective tissue cells produce IL-37 to prevent propagation of periodontal tissue destruction. Specifically, IL-37 is upregulated by gingival epithelial cells upon activation by disease typical PAMPs, which if excessive and prolonged may contribute to increase MMPs indicating a worse prognosis. We acknowledge that further experimental investigations are required to fully elucidate the exact mechanisms and confirm IL-37’s regulatory role in periodontal tissue protection.

## Data Availability

The datasets used and/or analysed during the current study are available from the corresponding author on reasonable request.

## Abbreviations

PAMP: Pathogen-associated molecular patterns
MMPs: Metalloproteinases
IL: Interleukin
LPS: Lipopolysaccharide
ELISA: Enzyme-linked immunosorbent assay
TLR: Toll-like receptor
GI: Gingival index
PD: Pocket depth
CAL: Clinical attachment level
BOP: Bleeding on probing
PI: Plaque index
PBS: Phosphate buffered saline
BSA: Bovine serum albumin
ANOVA: One-way analysis of variance
TIMP: Tissue inhibitors of metalloproteinase

## References

[1] Hajishengallis G. Periodontitis: from microbial immune subversion to systemic inflammation. Nat Rev Immunol. 2015;15(1):30–44. 25534621 10.1038/nri3785 PMC4276050

[2] Darveau RP. Periodontitis: a polymicrobial disruption of host homeostasis. Nat Rev Microbiol. 2010;8(7):481–90. 20514045 10.1038/nrmicro2337

[3] Lamont RJ, Koo H, Hajishengallis G. The oral microbiota: dynamic communities and host interactions. Nat Rev Microbiol. 2018;16(12):745–59. 30301974 10.1038/s41579-018-0089-x PMC6278837

[4] Curtis MA, Zenobia C, Darveau RP. The relationship of the oral microbiota to periodontal health and disease. Cell Host Microbe. 2011;10(4):302–6. 22018230 10.1016/j.chom.2011.09.008 PMC3216488

[5] Kinane DF, Stathopoulou PG, Papapanou PN. Periodontal diseases. Nat Rev Dis Primers. 2017;3(1):17038. 28805207 10.1038/nrdp.2017.38

[6] Graves DT, Corrêa JD, Silva TA. The oral microbiota is modified by systemic diseases. J Dent Res. 2019;98(2):148–56. 30359170 10.1177/0022034518805739 PMC6761737

[7] Pihlstrom BL, Michalowicz BS, Johnson NW. Periodontal diseases. Lancet. 2005;366(9499):1809–20. 16298220 10.1016/S0140-6736(05)67728-8

[8] Mäntylä P, Stenman M, Kinane D, Salo T, Suomalainen K, Tikanoja S, et al. Monitoring periodontal disease status in smokers and nonsmokers using a gingival crevicular fluid matrix metalloproteinase-8‐specific chair‐side test. J Periodontal Res. 2006;41(6):503–12. 17076774 10.1111/j.1600-0765.2006.00897.x

[9] Birkedal-Hansen H, Moore WGI, Bodden MK, Windsor LJ, Birkedal-Hansen B, DeCarlo A, et al. Matrix metalloproteinases: a review. Crit Reviews Oral Biology Med. 1993;4(2):197–250. 10.1177/10454411930040020401 8435466

[10] Beklen A, Tüter G, Sorsa T, et al. Gingival tissue and crevicular fluid co-operation in adult periodontitis. J Dent Res. 2006;85(1):59–63. 16373682 10.1177/154405910608500110

[11] Rakoff-Nahoum S, Paglino J, Eslami-Varzaneh F, Edberg S, Medzhitov R. Recognition of commensal microflora by toll-like receptors is required for intestinal homeostasis. Cell. 2004;118(2):229–41. 15260992 10.1016/j.cell.2004.07.002

[12] Medzhitov R, Horng T. Transcriptional control of the inflammatory response. Nat Rev Immunol. 2009;9(10):692–703. 19859064 10.1038/nri2634

[13] Abreu MT, Vora P, Faure E, Thomas LS, Arnold ET, Arditi M. Decreased expression of Toll-like receptor-4 and MD-2 correlates with intestinal epithelial cell protection against dysregulated Proinflammatory gene expression in response to bacterial lipopolysaccharide. J Immunol. 2001;167(3):1609–16. 11466383 10.4049/jimmunol.167.3.1609

[14] Socransky SS, Haffajee AD. Dental biofilms: difficult therapeutic targets. Periodontol 2000. 2002;28(1):12–55. 12013340 10.1034/j.1600-0757.2002.280102.x

[15] Barksby HE, Lea SR, Preshaw PM, Taylor J. The expanding family of interleukin-1 cytokines and their role in destructive inflammatory disorders. Clin Exp Immunol. 2007;149(2):217–25. 17590166 10.1111/j.1365-2249.2007.03441.x PMC1941943

[16] Dinarello CA. Interleukin-1 and the pathogenesis of the acute-phase response. N Engl J Med. 1984;311(22):1413–8. 6208485 10.1056/NEJM198411293112205

[17] Dinarello CA. Overview of the IL-1 family in innate inflammation and acquired immunity. Immunol Rev. 2018;281(1):8–27. 29247995 10.1111/imr.12621 PMC5756628

[18] Li Y, Wang Z, Yu T, Chen B, Zhang J, Huang K, et al. Increased expression of IL-37 in patients with graves’ disease and its contribution to suppression of Proinflammatory cytokines production in peripheral blood mononuclear cells. PLoS ONE. 2014;9(9):e107183. 25226272 10.1371/journal.pone.0107183 PMC4165889

[19] Zhan Q, Zeng Q, Song R, Zhai Y, Xu D, Fullerton DA, et al. IL-37 suppresses MyD88-mediated inflammatory responses in human aortic valve interstitial cells. Mol Med. 2017;23:83–91. 28362018 10.2119/molmed.2017.00022 PMC5429883

[20] Gao W, Kumar S, Lotze MT, Hanning C, Robbins PD, Gambotto A. Innate immunity mediated by the cytokine IL-1 homologue 4 (IL-1H4/IL-1F7) induces IL-12-dependent adaptive and profound antitumor immunity. J Immunol. 2003;170(1):107–13. 12496389 10.4049/jimmunol.170.1.107

[21] Keermann M, Kõks S, Reimann E, Abram K, Erm T, Silm H, et al. Expression of IL-36 family cytokines and IL-37 but not IL-38 is altered in psoriatic skin. J Dermatol Sci. 2015;80(2):150–2. 26319074 10.1016/j.jdermsci.2015.08.002

[22] Nold MF, Nold-Petry CA, Zepp JA, Palmer BE, Bufler P, Dinarello CA. IL-37 is a fundamental inhibitor of innate immunity. Nat Immunol. 2010;11(11):1014–22. 20935647 10.1038/ni.1944 PMC3537119

[23] Jing L, Kim S, Sun L, Wang L, Mildner E, Divaris K, et al. IL-37-and IL-35/IL-37-producing plasma cells in chronic periodontitis. J Dent Res. 2019;98(7):813–21. 31050915 10.1177/0022034519847443 PMC6589897

[24] Sağlam M, Köseoğlu S, Savran L, Pekbağriyanik T, Sağlam G, Sütçü R. Levels of interleukin-37 in gingival crevicular fluid, saliva, or plasma in periodontal disease. J Periodontal Res. 2015;50(5):614–21. 25399716 10.1111/jre.12241

[25] Papapanou PN, Sanz M, Buduneli N, et al. Periodontitis: consensus report of workgroup 2 of the 2017 world workshop on the classification of periodontal and Peri-Implant diseases and conditions. J Clin Periodontol. 2018;45(Suppl 20):S162–70. 29926490 10.1111/jcpe.12946

[26] Ebersole JL, Graves CL, Gonzalez OA, et al. Aging, inflammation, immunity and periodontal disease. Periodontol 2000. 2016;72(1):54–75. 27501491 10.1111/prd.12135

[27] Kiernan JA. Nuclear stains. Cold Spring Harb Protoc. 2008;2008(7):pdb–top50. 10.1101/pdb.top50 21356877

[28] Marsh PD, Devine DA. How is the development of dental biofilms influenced by the host? J Clin Periodontol. 2011;38:28–35. 21323701 10.1111/j.1600-051X.2010.01673.x

[29] Parahitiyawa NB, Scully C, Leung WK, Yam WC, Jin LJ, Samaranayake LP. Exploring the oral bacterial flora: current status and future directions. Oral Dis. 2010;16(2):136–45. 19627515 10.1111/j.1601-0825.2009.01607.x

[30] Kantrong N, To TT, Darveau RP. Gingival epithelial cell recognition of lipopolysaccharide. Oral Mucosal Immun Microbiome 2019;55–67. 10.1007/978-3-030-28524-1_5 31732934

[31] Lin B, Li R, Handley TNG, Wade JD, Li W, O’Brien-Simpson NM. Cationic antimicrobial peptides are leading the way to combat oropathogenic infections. ACS Infect Dis. 2021;7(11):2959–70. 34587737 10.1021/acsinfecdis.1c00424

[32] Sun Z, Ma L, Sin X, Sloan AJ, O’Brien-Simpson NM, Li W. The overview of antimicrobial peptide-coated implants againstoral bacterial infections. Aggregate. 2023;4:e309.

[33] Takeda K, Akira S. TLR signaling pathways. Seminars in immunology. Elsevier; 2004. pp. 3–9. 10.1016/j.smim.2003.10.003 14751757

[34] Kawasaki T, Kawai T. Toll-like receptor signaling pathways. Front Immunol. 2014;5:461. 25309543 10.3389/fimmu.2014.00461 PMC4174766

[35] Beklen A, Hukkanen M, Richardson R, Konttinen YT. Immunohistochemical localization of Toll-like receptors 1–10 in periodontitis. Oral Microbiol Immunol. 2008;23(5):425–31. 18793367 10.1111/j.1399-302X.2008.00448.x

[36] Henderson RB, Hobbs JAR, Mathies M, Hogg N. Rapid recruitment of inflammatory monocytes is independent of neutrophil migration. Blood. 2003;102(1):328–35. 12623845 10.1182/blood-2002-10-3228

[37] Xu WD, Zhao Y, Liu Y. Insights into IL-37, the role in autoimmune diseases. Autoimmun Rev. 2015;14(12):1170–5. 26264940 10.1016/j.autrev.2015.08.006

[38] Cavalli G, Dinarello CA. Suppression of inflammation and acquired immunity by IL-37. Immunol Rev. 2018;281(1):179–90. 29247987 10.1111/imr.12605

[39] Chen D, Wang Q, Ma Z, Chen F, Chen Y, Xie G, et al. MMP-2, MMP‐9 and TIMP‐2 gene polymorphisms in Chinese patients with generalized aggressive periodontitis. J Clin Periodontol. 2007;34(5):384–9. 17448043 10.1111/j.1600-051X.2007.01071.x

[40] Cazalis J, Tanabe S, ichi, Gagnon G, Sorsa T, Grenier D. Tetracyclines and chemically modified tetracycline-3 (CMT-3) modulate cytokine secretion by lipopolysaccharide-stimulated whole blood. Inflammation. 2009;32:130–7. 19238528 10.1007/s10753-009-9111-9

[41] Toraya S, Uehara O, Hiraki D, Harada F, Neopane P, Morikawa T, et al. Curcumin inhibits the expression of Proinflammatory mediators and MMP-9 in gingival epithelial cells stimulated for a prolonged period with lipopolysaccharides derived from Porphyromonas gingivalis. Odontology. 2020;108:16–24. 31087163 10.1007/s10266-019-00432-8

[42] Xie Y, Li Y, Cai X, Wang X, Li J. Interleukin-37 suppresses ICAM-1 expression in parallel with NF-κB down-regulation following TLR2 activation of human coronary artery endothelial cells. Int Immunopharmacol. 2016;38:26–30. 27233003 10.1016/j.intimp.2016.05.003

[43] Boraschi D, Lucchesi D, Hainzl S, Leitner M, Maier E, Mangelberger D, et al. IL-37: a new anti-inflammatory cytokine of the IL-1 family. Eur Cytokine Netw. 2011;22(3):127–47. 22047735 10.1684/ecn.2011.0288

[44] van Geffen EW, van Caam APM, van Beuningen HM, Vitters EL, Schreurs W, van de Loo FA, et al. IL37 dampens the IL1β-induced catabolic status of human OA chondrocytes. Rheumatology. 2017;56(3):351–61. 27940589 10.1093/rheumatology/kew411

[45] Pan Y, Wen X, Hao D, Wang Y, Wang L, He G, et al. The role of IL-37 in skin and connective tissue diseases. Biomed Pharmacother. 2020;122:109705. 31918276 10.1016/j.biopha.2019.109705

[46] Lee YS, Lan Tran HT, Van Ta Q. Regulation of expression of matrix metalloproteinase-9 by JNK in Raw 264.7 cells: presence of inhibitory factor (s) suppressing MMP-9 induction in serum and conditioned media. Exp Mol Med. 2009;41(4):259–68. 19299915 10.3858/emm.2009.41.4.029 PMC2679232

[47] Franco C, Patricia HR, Timo S, Claudia B, Marcela H. Matrix metalloproteinases as regulators of periodontal inflammation. Int J Mol Sci. 2017;18(2):440. 28218665 10.3390/ijms18020440 PMC5343974

[48] Dinarello CA, Nold-Petry C, Nold M, Fujita M, Li S, Kim S, et al. Suppression of innate inflammation and immunity by interleukin‐37. Eur J Immunol. 2016;46(5):1067–81. 27060871 10.1002/eji.201545828 PMC5003108

[49] Lorente L, Martin MM, Sole-Violan J, Blanquer J, Labarta L, Díaz C, et al. Association of sepsis-related mortality with early increase of TIMP-1/MMP-9 ratio. PLoS ONE. 2014;9(4):e94318. 24727739 10.1371/journal.pone.0094318 PMC3984125

[50] Mostafa Mtairag E, Chollet-Martin S, Oudghiri M, Laquay N, Jacob MP, Michel JB, et al. Effects of interleukin-10 on monocyte/endothelial cell adhesion and MMP-9/TIMP-1 secretion. Cardiovasc Res. 2001;49(4):882–90. 11230988 10.1016/s0008-6363(00)00287-x

